# Gold Coated Lanthanide Phosphate Nanoparticles for Targeted Alpha Generator Radiotherapy

**DOI:** 10.1371/journal.pone.0054531

**Published:** 2013-01-18

**Authors:** Mark F. McLaughlin, Jonathan Woodward, Rose A. Boll, Jonathan S. Wall, Adam J. Rondinone, Stephen J. Kennel, Saed Mirzadeh, J. David Robertson

**Affiliations:** 1 Department of Chemistry and University of Missouri Research Reactor, University of Missouri, Columbia, Missouri, United States of America; 2 Oak Ridge National Laboratory, Oak Ridge, Tennessee, United States of America; 3 Graduate School of Medicine, University of Tennessee, Knoxville, Tennessee, United States of America; Vanderbilt University, United States of America

## Abstract

Targeted radiotherapies maximize cytotoxicty to cancer cells. *In vivo* α-generator targeted radiotherapies can deliver multiple α particles to a receptor site dramatically amplifying the radiation dose delivered to the target. The major challenge with α-generator radiotherapies is that traditional chelating moieties are unable to sequester the radioactive daughters in the bioconjugate which is critical to minimize toxicity to healthy, non-target tissue. The recoil energy of the ^225^Ac daughters following α decay will sever any metal-ligand bond used to form the bioconjugate. This work demonstrates that an engineered multilayered nanoparticle-antibody conjugate can deliver multiple α radiations and contain the decay daughters of ^225^Ac while targeting biologically relevant receptors in a female BALB/c mouse model. These multi-shell nanoparticles combine the radiation resistance of lanthanide phosphate to contain ^225^Ac and its radioactive decay daughters, the magnetic properties of gadolinium phosphate for easy separation, and established gold chemistry for attachment of targeting moieties.

## Introduction

Beta emitting radionuclides have found widespread use in cancer therapy. A major advance in nuclear medicine was the development of targeted endo-radiotherapies with two targeted radiotherapy agents approved for clinical use. BEXXAR®, labeled with ^131^I, is used to treat follicular lymphoma while Zevalin®, containing ^90^Y, is used for treatment of B cell non-Hodgkins lymphoma [1]–[2]. Other targeted radiotherapy agents labeled with β^−^ emitters ^131^I, ^90^Y, ^177^Lu, and ^188^Re are showing promise in ongoing clinical trials [3]–[4]. One of the challenges associated with β^−^ emitting targeted radionuclide therapies is, however, the inherent toxicity from the death of normal, healthy cells resulting from the crossfire radiation damage from the relatively long ranges of the β^−^ particles in tissue [5]. For example, β^−^ particles from ^177^Lu (β_max_ = 0.5 MeV) have a range of 1.5 mm in tissue and β^−^ particles from ^90^Y (β_max_ = 2.3 MeV) deposit their energy over a range of 12 mm. Targeted radiotherapies based on α particles are a promising alternative to β^−^ particles because the α particles deposit all of their energy within a few cell diameters (50–100 µm). Because of their much shorter range, targeted α-radiotherapy agents have great potential for application to small, disseminated tumors and micro metastases and treatment of hematological malignancies consisting of individual, circulating neoplastic cells [6]. Compared with β^−^ particles, α particles provide a very high relative biological effectiveness, killing more cells with less radioactivity. The high linear energy transfer of α particles induces significantly more DNA double strand breaks than β^−^ particles [7]. Also, the biological effectiveness of α particles does not depend upon hypoxia or cell cycle considerations [8]–[9]. Most α emitters also have a relatively low γ-ray component in their decay allowing for outpatient treatments and lower radiation doses to nuclear medicine personnel [10].

**Figure 1:**
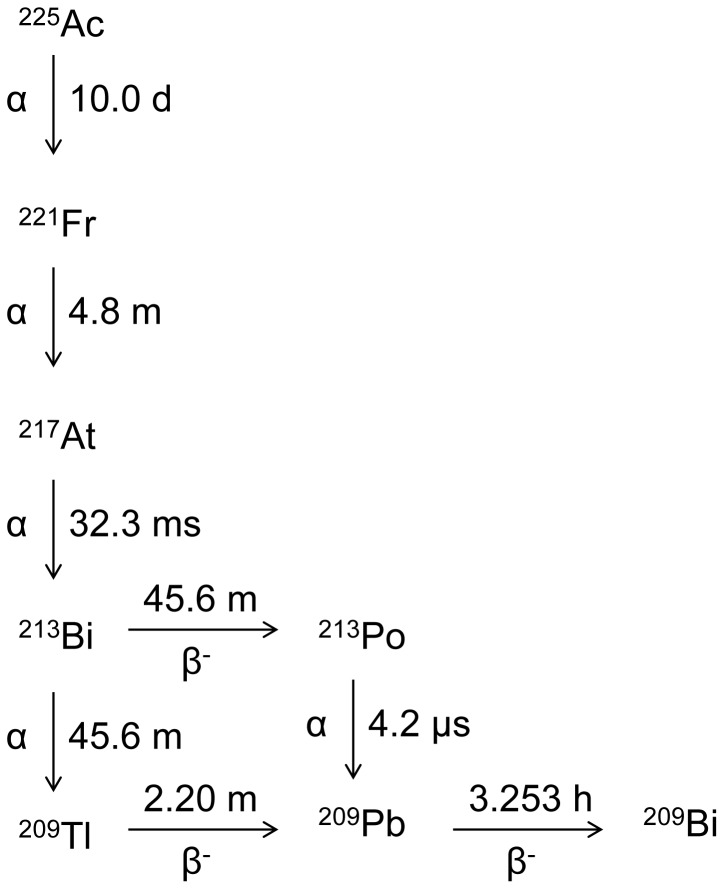
Abbreviated decay scheme of ^225^Ac. ^225^Ac emits 4 α particles in the process of decaying to the long-lived ^209^Bi.

A number of targeted alpha therapy (TAT) agents based on the single alpha emitting radionuclides ^211^At (t_1/2_ = 7.2 h), ^213^Bi (t_1/2_ = 46 m),^ 212^Pb (t_1/2_ = 10.6 h), and ^212^Bi (t_1/2_ = 61 m) have been developed and are showing promise in pre-clinical and clinical trials [11]. The radiotherapeutic efficacy of TAT could, however, be further enhanced by use of *in vivo* α-generator radionuclides like ^225^Ac, which emits four α particles in its decay chain (Figure 1). The median lethal dose of ^225^Ac constructs is one to two orders of magnitude lower than the LD_50_ values for the corresponding single α emitting ^213^Bi labeled antibodies *in vitro* with a number of cancer cell types [12]. Moreover, the longer half-life of ^225^Ac (t_1/2_ = 10 d) reduces activity loss during radiopharmaceutical synthesis and allows greater time for localization of antibodies to receptor sites.

Despite these advantages, there is a distinct challenge associated with targeted *in vivo* α-generator radiotherapy. If the α-emitting daughter products in the ^225^Ac decay chain are not sequestered at the target site, they can migrate and deliver a potentially toxic dose to non-target tissue [11]. The recoil energy of the ^225^Ac daughters following alpha decay (>100 keV) will sever any metal-ligand bond used to form the bioconjugate, releasing the daughter radionuclides from the targeting agent. Renal toxicity is currently the dose-limiting factor in clinical use of ^225^Ac. In the recent work of Schwartz et al., almost 80% of the absorbed dose to the renal medulla was delivered by free ^213^Bi when using a metal-ligand bioconjugate to deliver ^225^Ac in a mouse model [13]. Metal-ligand bioconjugates fail to sequester the daughter products of ^225^Ac (^221^Fr, ^217^At, and ^213^Bi) and the released ^213^Bi accumulates in the kidney [14]–[15]. An alternative strategy to this challenge, incorporating ^225^Ac in engineered liposomes, was found to retain less than 10% of the ^213^Bi activity from the decay of ^225^Ac *in vitro* [16].

The *in vivo* α generator ^223^Ra, which also emits four alpha particles in its decay chain, is an effective treatment for metastatic bone cancer [17]. Radium-223 chloride has been granted Fast Track designation by the U.S. Food and Drug Administration for the treatment of hormone-refractory prostate cancer in patients with bone metastases [18]. It is effective in this case because radium is a calcium mimic with a high affinity for bone tissue and the daughter products either have short half-lives or have a high affinity for bone (^211^Pb, t_1/2_ = 36 m). Translation of *in vivo* α generators to anything besides metastatic bone cancer with a calcium mimic will require a different mechanism for both delivering and retaining the radioactive daughters in the target tissue.

**Figure 2:**
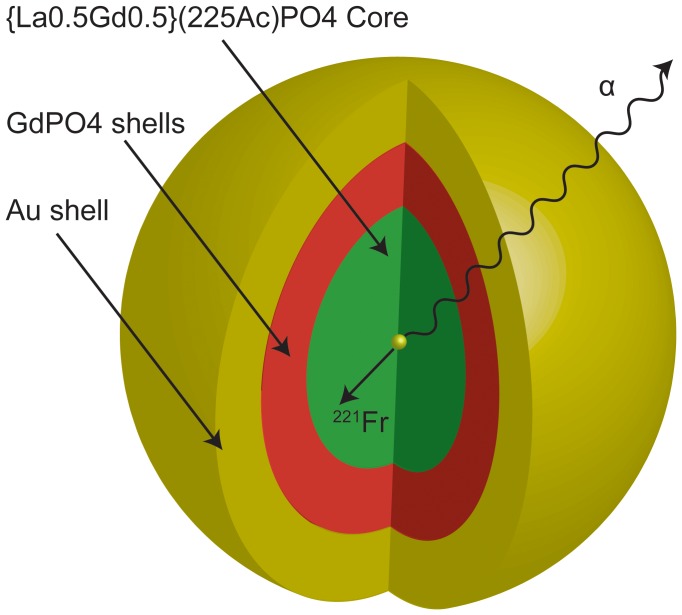
Schematic of gold coated lanthanide phosphate NP. The α emitter is loaded in the {La_0.5_Gd_0.5_}PO_4_ core, the GdPO_4_ layer(s) increase retention of the decay chain daughters, and the Au shell facilitates attachment of targeting agents.

In this work, we demonstrate that a multilayered nanoparticle (NP) can contain the recoiling daughters of the *in vivo* α generator, and when coupled to a targeting antibody, can bind to biologically relevant receptors and deliver multiple α particles to each receptor site. While the range of the recoiling daughters is less than the diameter of the layered NPs, the α-particles lose less than 0.2% of their energy as they exit from the center of the NPs. The layered NPs consist of ^225^Ac-doped {La_0.5_Gd_0.5_}PO_4_@GdPO_4_@Au. A schematic of the layered NP design is illustrated in Figure 2. These multi-shell particles combine the radiation resistance of crystalline lanthanide phosphate [19], [20]–[21] to encapsulate and contain atoms of the therapeutic radionuclide ^225^Ac and its radioactive daughters, the magnetic properties of gadolinium phosphate NPs, and established surface chemistry of gold NPs for attachment of targeting agents [22].

Nanoparticles offer a number of favorable properties with regard to drug delivery. The ability to hold multiple copies of a therapeutic or imaging moiety provides the ability to generate efficacious results even against targets with low receptor numbers *in vivo* [23]. Multi-functional, layered NPs allow for synergistic combinations of properties exhibited by the individual NPs including containment, purification and conjugation. Moreover, in the system described in this work, GdPO_4_ could function as a magnetic resonance imaging (MRI) contrast agent [24] and Au can be doped with the single photon emission computed tomography (SPECT) radionuclide ^199^Au for γ-ray imaging [25].

## Results and Discussion

{La_0.5_Gd_0.5_}(^225^Ac)PO_4_ core particles were synthesized by hydrolysis of sodium tripolyphosphate (Na-TPP) in the presence of equimolar mixtures of La and Gd salts with ^225^Ac present at the tracer level. Nanoparticles of diameter *ca.* 4 nm precipitated out of solution after heating for 3 hours at 90°C. In order to improve retention of the decay daughters, four additional shells of pure GdPO_4_ were added, each by dispersing the precipitated NPs in a solution of Gd^3+^ and Na-TPP and heating for an additional 3 hours. Gold was then added to the NPs by reduction of NaAuCl_4_ with sodium citrate.

**Figure 3:**
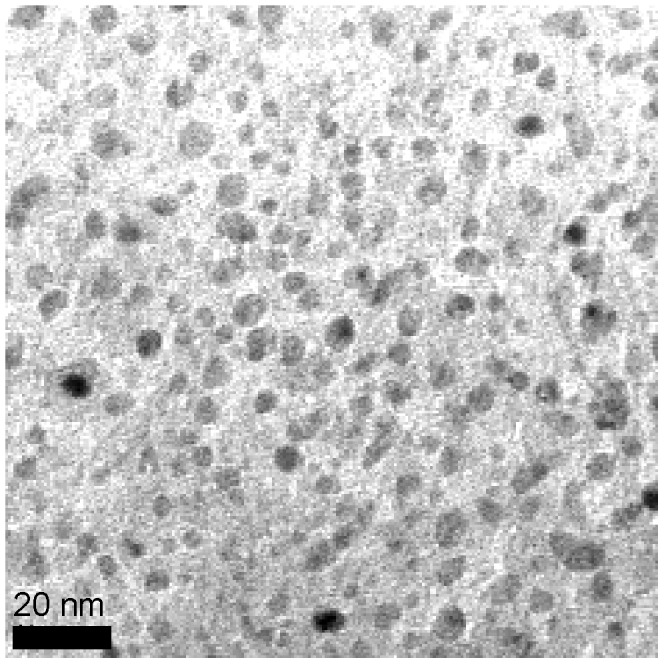
TEM image of {La_0.5_Gd_0.5_}PO_4_ core NPs.

Rietveld refinement of x-ray diffraction (XRD) patterns indicated that LaPO_4_ NPs exhibited the rhabdophane phase consistent with the description of Buissette *et al.* [26]. However, the {La_0.5_Gd_0.5_}PO_4_, {La_0.25_Gd_0.75_}PO_4_, and GdPO_4_ systems crystallized in the anhydrous monazite phase [27]. The monazite phase for LaPO_4_ NPs was previously observed for crystalline synthesis in organic solvents [28]. The XRD measurements yielded NP grain sizes of 4.04 nm for LaPO_4_, 2.79 nm for {La_0.5_Gd_0.5_}PO_4_, 2.91 nm for {La_0.25_Gd_0.75_}PO_4_ and 3.11 nm for GdPO_4_. Size estimates of the {La_0.5_Gd_0.5_}PO_4_ NPs from transmission electron microscopy (TEM) images (Figure 3) match the grain sizes predicted by XRD, indicating that the core particles were a single crystal phase. Neutron activation analysis of magnetically separated {La_0.5_Gd_0.5_}PO_4_ core NPs gives a La to Gd mole ratio of 1.11±0.03. Pure LaPO_4_ and pure GdPO_4_ exhibited larger grain sizes than their mixed counterparts.

**Figure 4:**
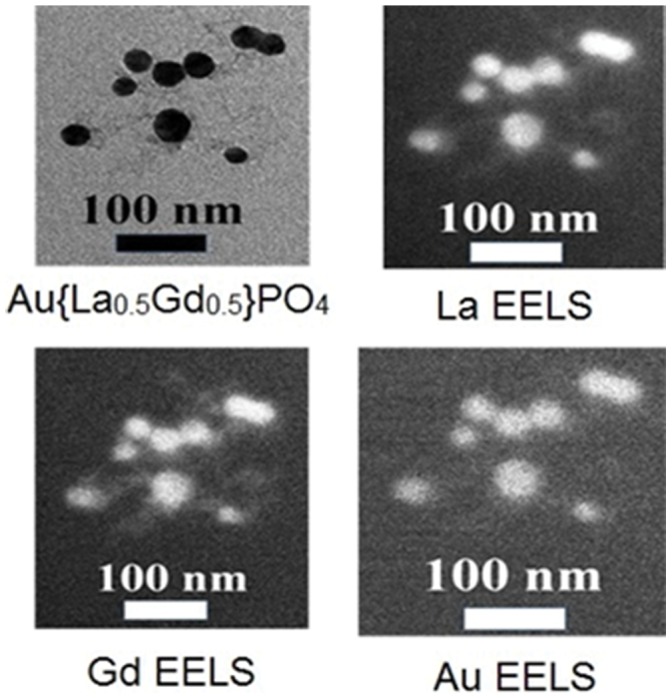
TEM of a characteristic cluster of NPs. EELS analysis indicates the presence of La, Gd, and Au in all particles in the cluster.

Addition of GdPO_4_ shells to the core {La_0.5_Gd_0.5_}PO_4_ NP causes epitaxial growth of the particle. Mean diameters increase sequentially with each shell addition (Table 1). Addition of four GdPO_4_ shells to the core {La_0.5_Gd_0.5_}PO_4_ produces 22 nm diameter NPs and addition of an outer gold layer increases the particle diameter to 27 nm. Electron energy loss spectroscopy (EELS)-TEM images of the NPs are shown in Figure 4.

**Table 1:** Growth of NP diameter as a function of shell addition as measured by TEM.

Particle System	Diameter (nm)
{La_0.5_Gd_0.5_}PO_4_ Core	5.0±1.5
{La_0.5_Gd_0.5_}PO_4_@1 shell GdPO_4_	7.8±2.8
{La_0.5_Gd_0.5_}PO_4_@2 shells GdPO_4_	9.9±2.6
{La_0.5_Gd_0.5_}PO_4_@3 shells GdPO_4_	13.3±1.8
{La_0.5_Gd_0.5_}PO_4_@4 shells GdPO_4_	22.4±7.7
{La_0.5_Gd_0.5_}PO_4_@4 shells GdPO4@Au	26.8±4.9

Gold coated NPs with four epitaxially added GdPO_4_ shells were further characterized by dynamic light scattering. Hydrodynamic diameters and zeta potentials are shown in Table 2. An increase of the hydrodynamic diameter on addition of polyethylene glycol (PEG) and antibody is common for NPs. The highly negative zeta potentials should lead to stability in water which was confirmed by monitoring changes in the UV-Vis spectrum of the particles over a 1 month period in both 18 MΩ water and saline solution. No shift was observed in the plasmon resonance over this time period.

**Table 2:** Dynamic light scattering of NPs in 18 MΩ water.

Particle	Hydrodynamic diameter (nm)	Zeta potential (mV)
{La_0.5_Gd_0.5_}(^225^Ac)PO_4_@GdPO_4_@Au	101.4±1.5	−63.2±1.6
{La_0.5_Gd_0.5_}(^225^Ac)PO_4_@GdPO_4_@Au-PEG	382.3±6.5	−56.4±0.1
{La_0.5_Gd_0.5_}(^225^Ac)PO_4_@GdPO_4_@Au-mAb-201b	1498±77	−27.9±2.4

Nanoparticles with GdPO_4_ shells followed by Au coating dramatically increased radioactive daughter retention *in vitro* compared with previously published results for core-only lanthanum phosphate NPs [28]. Adding 2 shells increased retention of the ^221^Fr daughter from 50% for the LaPO_4_ core to 70%. With four shells of GdPO_4_, the initial retention of the ^221^Fr daughter was 98%. Daughter retention decreased by roughly 2% per day over the course of a week, and stabilized at 88%. Further, the presence of the Au/4 GdPO_4_ shells increased the retention of the ^225^Ac parent itself by roughly an order of magnitude. Over the course of 3 weeks, the multi-layered particles retained greater than 99.99% of the ^225^Ac parent radionuclide. Particles with more than 4 shells of GdPO_4_ settled out of solution rapidly and were difficult to manipulate. Monitoring the plasmon resonance indicated that the multi-layered particles remained stable towards aggregation in PBS over the course of one month.

**Figure 5:**
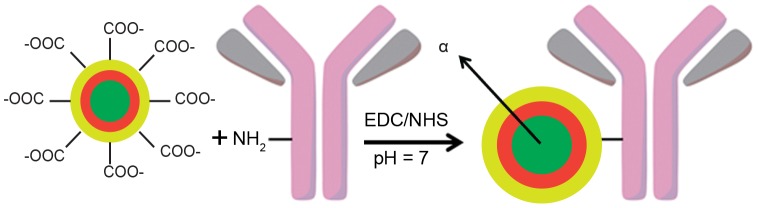
MAb 201b antibody conjugation to multi-layered NPs.

For *in vivo* biodistribution testing, the NPs were conjugated to the mAb 201b monoclonal antibody via a lipoic acid-PEG_12_-COOH linker [29]. MAb 201b targets thrombomodulin receptors which are highly expressed in lung endothelium. The antibody quickly localizes to its vascular target and clears from circulation with a half-life of 40 hours [30]. 3-sulfo-N-hydroxysuccinimide (sulfo-NHS) and 1-ethyl-3-(3-dimethylaminopropyl)-carbodiimide (EDC) activated the carboxylate of the PEG for coupling to amine groups on the antibody, leading to the formation of an amide bond. The reaction was quenched with glycine and conjugates were purified by centrifugation. The conjugated NPs were redispersed in phosphate buffered saline (PBS) containing bovine serum albumin (BSA). The antibody conjugation process is summarized in Figure 5.

**Figure 6:**
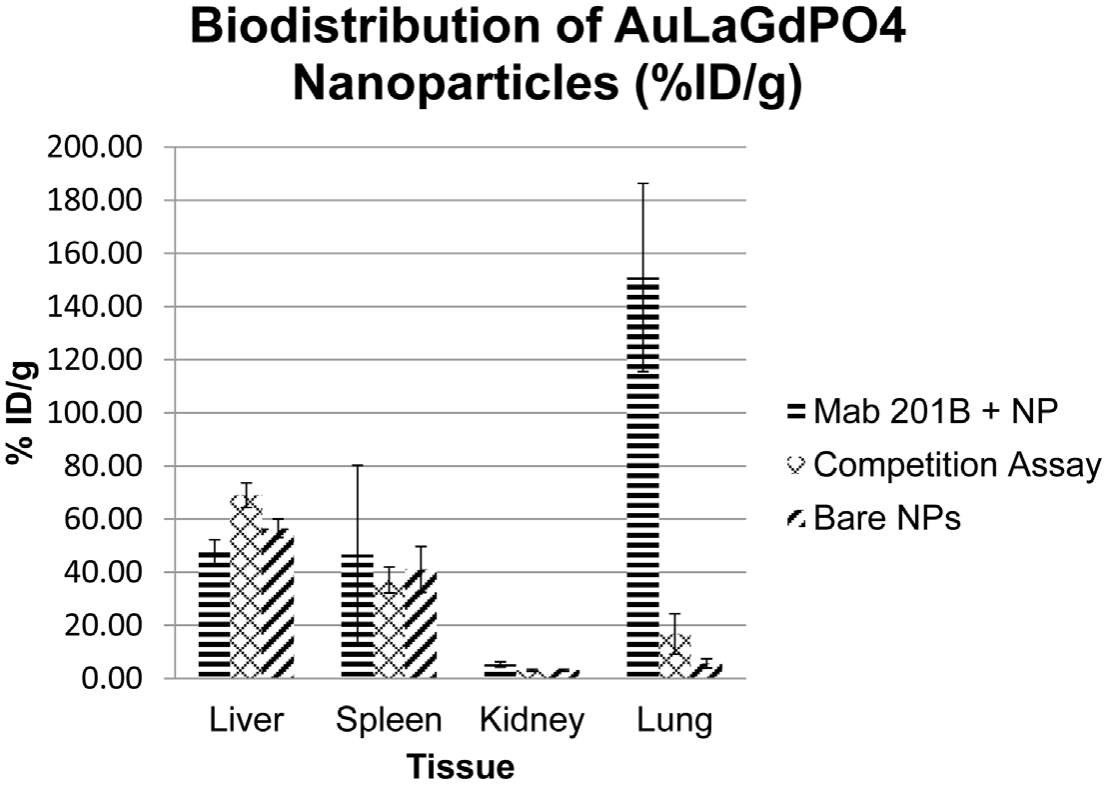
Biodistribution of NPs following tail vein injection in mice at 1 hour (n = 3).

*In vivo* biodistribution experiments of the ^225^Ac containing NPs (*ca.* 2 µCi/animal) demonstrated that the antibody-targeted NPs localized in the lung consistent with the binding properties of mAb 201b. The NPs exhibit high lung uptake with the antibody conjugate after 1 hour (151%ID/g). This high lung uptake dropped to 16.8%ID/g when the antibody conjugated NPs were competed with unconjugated antibody (Figure 6). These results demonstrate that the antibody retained its binding affinity and specificity even after conjugation to the NPs and that the NPs localized in the lung through antibody binding. While the antibody-labeled NPs cleared rapidly from the lungs in these proof-of-principle experiments (after 24 hours, ^225^Ac activity was predominantly present in the liver and spleen), previous strategies used to reduce reticuloendothelial functioning such as treatment with clodronate liposomes could be applied to mitigate the rapid clearance [31], [32]–[33].

Retention of ^213^Bi, from the decay of ^225^Ac in the α-generator NPs, was 69% ±3% in lung tissue after 1 hour and increased to 84% ±3% after 24 hours. Similar ^213^Bi retention values were observed in liver (1 h, 81% ±4%; 24 h, 92% ±1%) and spleen tissue (1 h, 72% ±3%; 24 h, 82% ±16%). Despite the widespread renal toxicity concerns associated with ^213^Bi relocation to the kidney from ^225^Ac α-generator therapies, only 2.8% of the ^213^Bi from the injected dose migrated to kidney tissues after 1 hour. After 24 hours, this number further decreased to 1.5%.

**Figure 7:**
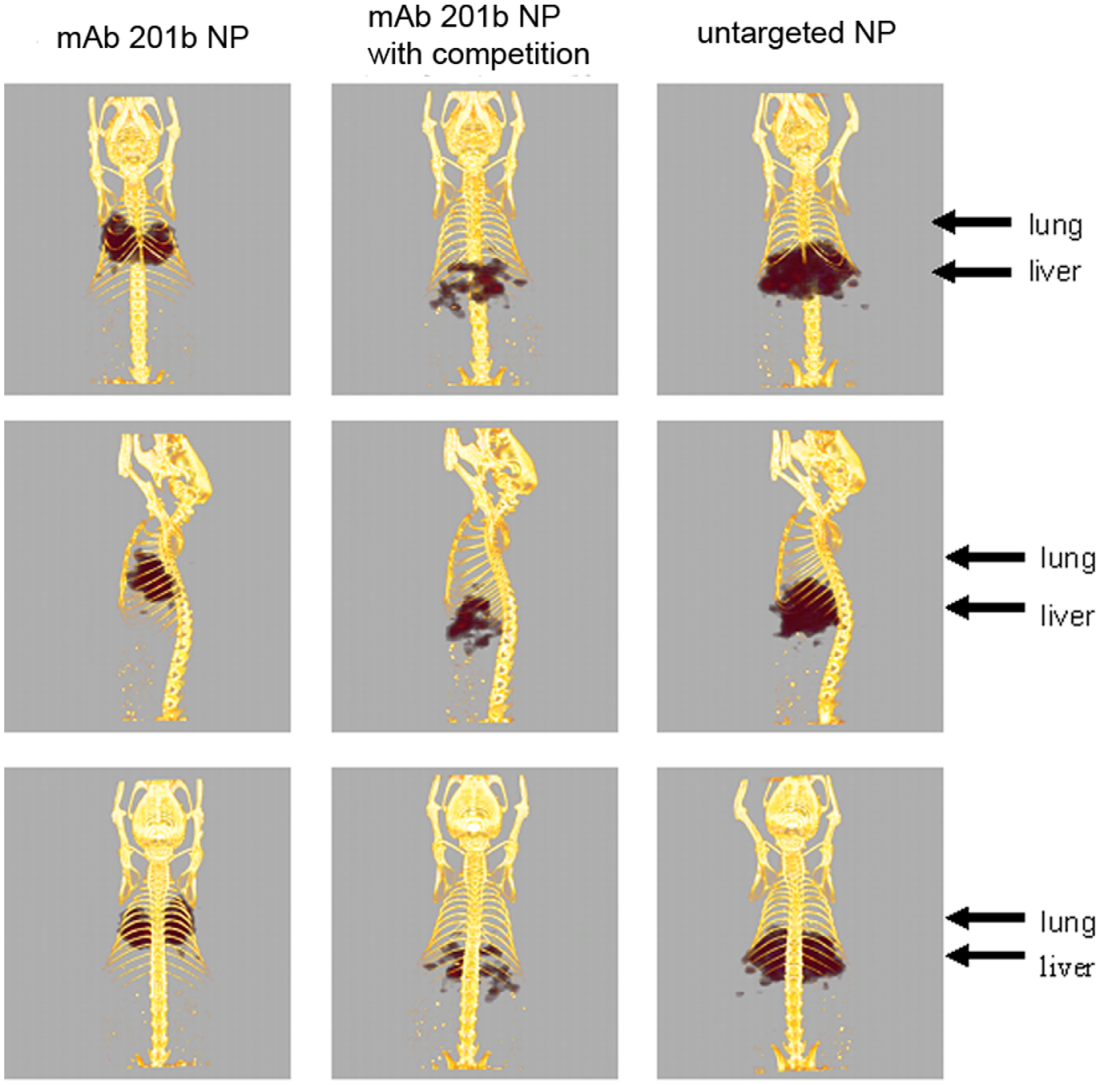
SPECT/CT images 1 hour post-injection of 80 µCi of {La_0.5_Gd_0.5_}(^225^Ac)PO_4_@GdPO_4_@Au-mAb-201b.

A larger dose (*ca.* 80 µCi/animal) of ^225^Ac NPs was imaged using CT/SPECT of the ^221^Fr γ-ray (218 keV, 11.6%). Mice injected with this larger dose were sacrificed 1 hour post-injection and imaged 3 hours post-sacrifice to allow the daughter products of ^225^Ac to reach their equilibrium activities. The CT/SPECT images (Figure 7) clearly show large uptake in the lung for the {La_0.5_Gd_0.5_}(^225^Ac)PO_4_@GdPO_4_@Au-mAb-201b NPs which is in agreement with the biodistribution data. When competed with unconjugated mAb 201b antibody, the images showed high uptake in the liver. If the antibody conjugated NPs cannot bind their *in vivo* target, they are cleared from circulation via the reticuloendothelial system. Finally, PEG coated NPs without antibody also show high uptake in the reticuloendothelial system (Figure 7), further indicating that the lung uptake is not due to particulate trapping in the small capillary system.

{La_0.5_Gd_0.5_}(^225^Ac)PO_4_@GdPO_4_@Au NPs represent a novel system for targeted α radiotherapy. Adding a Au surface onto a LnPO_4_ core (Ln = La, Gd) allows for facile, reproducible surface functionalization. The addition of Gd into the particles creates a magnetic moment which is sufficient to separate the gold NPs containing Gd from any gold NPs produced in the gold coating step. This separation ensures that gold NPs without a radioactive core will not compete with the TAT conjugate for receptor sites.

Compared with single α-emitting therapies, the use of *in vivo* α generators holds the potential to deliver a much larger biologically effective dose to target tissues. Effective design of *in vivo* TAT agents with isotopes like ^225^Ac requires two major components. First, the therapeutic agent must be able to deliver the generator radionuclide specifically to target tissue at a cytotoxic dose. The high, receptor-mediated uptake of particles in the lung endothelium demonstrates the ability of {La_0.5_Gd_0.5_}(^225^Ac)PO_4_@GdPO_4_@Au NPs to deliver ^225^Ac to a tissue target that is present in the vascular space. Second, the TAT must be able to retain the daughter products of the generator in the target tissue. Migration of daughter products to non-target tissue will severely limit the administered therapeutic dose. Retention of the decay daughters can be achieved in a number of ways. First, the radionuclide may be selected so that the daughter half-lives are sufficiently short that they will not have time to migrate throughout the body. Alternatively, the radionuclide can be chosen so that the daughter products exhibit similar *in vivo* behavior and remain in the target tissue. This is the principle behind the recent successes using ^223^RaCl_2_ for treatment of bone metastases [34]. The ^223^Ra daughter products either have short half-lives or have a high affinity for bone (^211^Pb, t_1/2_ = 36 m). While effective in this case, translation of this *in vivo* α generator to other tumor types would require a different mechanism of retaining the ^211^Pb and ^211^Bi daughters in the target tissue. A third solution to the daughter retention problem involves internalization of the parent radionuclide in the target cell itself [12]. This approach utilizes the internal milieu of the cell to contain the daughter decay products. Tumor targets for internalization occur largely in the extravascular space, which is difficult to access with larger constructs that promote endocytosis. Attempts to reduce ^213^Bi toxicity through targeted, metal-chelate based internalizing antibodies have shown only moderate success [35].

The NP construct described in this work improves ^225^Ac daughter retention relative to both chelate approaches and previous NP constructs. {La_0.5_Gd_0.5_}(^225^Ac)PO_4_@GdPO_4_@Au NPs contain 88% of the ^221^Fr daughter *in vitro*, compared with 50% retention observed with La(^225^Ac)PO_4_ NPs [28]. Additionally, the *in vivo* α-generator delivery agent has a negligible effect on the energies of the emitted α particles. A 6 MeV α-particle loses less than 0.2% of its energy in the layered NP whereas the range of the 100 keV recoiling daughters is ∼20 nm in bulk LnPO_4_. Moreover, a portion of the kinetic energy of the daughter particle may be transferred to the entire particle. If a portion of the recoil energy is distributed throughout the highly structured crystalline lattice, the recoiling range of the daughter radionuclides will be significantly decreased [36].

*In vivo*, the increase of retention of ^213^Bi in the target tissue over time results from a combination of the ability of the layered NPs to retain the daughter products and endocytosis of the TAT NP. In this work, ^213^Bi daughter retention *in vivo* with the layered NP showed improvement over the LaPO_4_ core NP [28]. The ^213^Bi retention is lower than the ^221^Fr retention because prior decays of ^225^Ac, ^221^Fr, and ^217^At can move the remaining α-emitting nuclides towards the surface of the NP. From this position nearer the surface, subsequent α decays are likely to release the daughter nuclide from the NP. The amount of ^213^Bi which relocates to the kidney from other tissues shows marked improvement with the {La_0.5_Gd_0.5_}(^225^Ac)PO_4_@GdPO_4_@Au-mAb-201b system compared with the La(^225^Ac)PO_4_-mAb-201b system.^27^ Only 2.8% of the injected dose migrated to the kidney as ^213^Bi after 1 hour and 1.5% after 24 hours in the layered NPs while 10% of the ID relocated to the kidney after 1 hour and 5% after 24 hours with the core only lanthanum phosphate NPs.

These experiments demonstrate that multi-functional, layered NPs can be used to deliver and retain ^225^Ac and its daughter radioisotopes at a target site thereby reducing the absorbed dose to non-target organs. TAT experiments in a model tumor system are in progress to directly assess the efficacy of the constructs.

## Materials and Methods

All chemicals were used as received from Sigma-Aldrich and were at least ACS grade unless otherwise noted. Water originated from an in house 18 MΩ MilliQ system. Radioactivity measurements were performed with γ-ray spectroscopy employing a calibrated high purity germanium detector employing a PC-based multichannel analyzer (Canberra Industries) windowed on ^221^Fr (212 keV) and ^213^Bi (440 keV). ^225^AcCl_3_ was prepared as previously described from a ^229^Th cow [28]. A Spectra/Por 10 kDa molecular weight cutoff (MWCO) regenerated cellulose dialysis membrane was used to separate NPs from solutions. Dialysis membranes were washed of preservatives before use against 18 MΩ water. A large NdFeB magnet (3″ O.D.×0.5″ thick, surface field = 0.4 T) was obtained from United Nuclear.

### Preparation of {La_0.5_Gd0_.5_(^225^Ac)}PO_4_ Core Particles

Core particles were made by modifying a methodology developed by Buissette *et al*. [26]. Briefly, 50 µL each of 0.1 M LaCl_3_ and GdCl_3_ were mixed in a 1 mL V-bottom vial with spin vane. For the synthesis of radioactive NPs, 5.2 mCi of ^225^AcCl_3_ in 0.1 M HCl was added to the lanthanide mixture. Next, 200 µL of 0.1 M sodium tripolyphosphate (Na-TPP) was added to give a total Ln:Na-TPP ratio of 1∶2 resulting in a clear, colorless solution. If the solution remained turbid after addition of Na-TPP, it was vortexed with small (10 µL) additions of Na-TPP until the solution appeared clear. The resulting solution was then capped and heated at 90°C for 3 hours giving a turbid, white suspension of particles. Particles were purified via dialysis overnight. This preparation produced monodisperse particles of ∼4 nm diameter which were characterized by transmission electron microscopy (TEM, JEOL 1400), neutron activation analysis (NAA) and x-ray diffraction (XRD, Scintag X2).

### Layering of Particles

Core particles described above were centrifuged at 3,000 g for 3 minutes and the supernatant was removed. The particles were redispersed in a solution consisting of 200 µL of 0.05 M GdCl_3_ and 400 µL 0.05 M Na-TPP. The resulting mixture was vortexed briefly then sonicated using a bath sonicator for 10 minutes before heating at 90°C for three hours. This process was repeated for up to four shell additions, at which point the solution becomes a thick milky white. Particles were purified by dialysis as above before gold coating.

The dialyzed particles (12 mg) were collected and split evenly between three 5-mL V-bottom vials. 300 µL of 0.1 M tribasic sodium citrate was added to each vial along with 1.5 mL of 18 MΩ water. Next, 2.5 mL of 1 mM NaAuCl_4_^−^ was added dropwise to the solution slowly at the rate of 1 mL every 10 minutes. After the final addition, the solution was kept at 90^0^C for 30–45 minutes. A large NdFeB magnet (surface field = 0.4 T) was placed next to the V-bottom vial for 16 hours to separate the particles from solution. The supernatant was decanted to isolate the magnetically active particles. In the radiotracer labeling experiments, the separation efficiency was determined by γ-ray spectrometry of the removed supernatant and magnetically collected particles [28]. Non-radioactive analogs of the particles were characterized by EELS-TEM (Zeiss Libra 120) and NAA.

### *In Vitro* Testing of ^221^Fr Retention

In order to test retention of the ^225^Ac decay products *in vitro*, the ^225^Ac-NPs were loaded into a dialysis membrane and dialyzed against 400 mL of 18 MΩ water. The dialysis tube was stirred for a sufficient time for daughter equilibrium to be established (>3 hours), then a 5 mL aliquot was taken for γ-ray spectrometry analysis. Each sample was re-analyzed at a later time to determine the level of ^225^Ac in the removed dialysate fraction. The measured activities were corrected for decay and dialysate loss from prior aliquot removals. The ^213^Bi activity in the dialysate was used as a measure of the ^221^Fr that was released from the NP, as ^213^Bi which escaped from the particles did not move across the dialysis membrane [28].

### Surface Modification

Surfaces were modified using a lipoamide-dPEG_12_-acid linker (Quanta Biodesign). Two mg of dPEG were added, followed by 6 mg of Tris(2-carboxyethyl)phosphine reducing agent to cleave the disulfide bond. The pH of the solution was adjusted to 7 using 0.1 M NaOH and the reaction mixture was stirred for 4 hours. Connection of the linker was determined by a shift in the plasmon resonance near 530 nm as monitored by UV-Vis spectroscopy before and after the addition of the linker.

### Antibody Conjugation

To attach antibodies to the linker, the carboxylate group on the PEG linker was first activated using 8 µL of 10 mg/mL aqueous 3-sulfo-N-hydroxysuccinimide (sulfo-NHS) and 80 µL of 10 mg/mL 1-ethyl-3-(3-dimethylaminopropyl)-carbodiimide (EDC). 4 mg of EDC and 0.4 mg of sulfo-NHS were used per mg of NPs. After 15 minutes, the solutions were centrifuged to remove excess EDC/NHS, the supernatant removed, and the particles redispersed in phosphate buffered saline (0.01 M NaPO_4_ pH 7.6 in 0.15 M NaCl, PBS). MAb 201b was added (∼ 1 mg of mAb/mg of NP), and the mixture was mixed by rotation overnight. The reaction was then quenched with 1 mg of glycine, which was allowed to react for 15 minutes. The particles were then centrifuged to remove excess antibody. The supernatant was removed and the particles were redispersed in PBS containing 5 mg/mL bovine serum albumin (BSA/PBS). The particles were sonicated with a Branson microprobe for 10 sec and vortexed prior to injection. The final product of the mAb conjugated NP was ∼3 mg/mL NP with 400 µCi of ^225^Ac and ∼1 mg mAb 201b.

### Biodistribution Studies

All experiments involving mice were performed according to the Institutional Animal Care and Use Committee of the University of Tennessee approved protocol 1502. Female BALB/c mice (body mass ∼20 g) were used for all biodistribution and imaging experiments. Biodistribution and daughter retention assays were done on three groups, consisting of three mice per group, were each injected intravenously (tail vein). Groups 1 and 2 were injected with Au/GdPO_4_/{La_0.5_Gd_0.5_(^225^Ac)}PO_4_-mAb-201b outer shell/inner shell/core conjugates, while group 3 was treated with Au/GdPO_4_/{La_0.5_Gd_0.5_(^225^Ac)}PO_4_-PEG NPs as a control. Group 1 mice received 14.6 µg of NP with 1.95 µCi of Ac-225 and ∼ 5 µg of attached mAb 201b (this value was estimated from data in a parallel experiment wherein about 30% of added radioiodinated mAb was incorporated in NP under similar conditions). Group 2 received the same amount of targeted NP but with the addition of 750 µg of free mAb 201b as competitor. Group 3 received the same amount of NP and Ac-225, but with no targeting agent conjugated. Mice were housed with food and water ad libitum in a light/dark cycle environment before sacrificing at 1 and 24 h post-injection for biodistribution and *in vivo* retention studies. Biodistribution studies were performed on lungs, liver, spleen, and kidneys to evaluate the amount of both ^221^Fr and ^213^Bi in target organs by measuring weighed tissue samples in a γ-ray scintillation counter at a specific time postsacrifice and again after the radioisotopes had achieved decay equilibrium (>3 h). Quantities of ^221^Fr and ^213^Bi present at the time of animal sacrifice were determined by appropriate crossover and decay corrections as previously described [28].

### MicroSPECT/CT Imaging

Small animal imaging was performed using a microCAT IIþ SPECT dual modality platform (Siemens Preclinical Imaging, Knoxville, TN). Mice were injected with approximately 40 times more NP than were the mice for biodistribution studies. Thus the mice for competition with cold mAb 201b did not have the same ratio of cold competitor to radiolabeled NP and competition was not as complete.Animals were sacrificed by overdose of isoflurane at 1 h postinjection and imaged via microSPECT/CT 3 h later when the ^225^Ac and its daughters had reached equilibrium. SPECT data for the final images were acquired as previously described by Woodward *et al.* [28].

## References

[1] Jacene HA, Filice R, Kasecamp W (2007) Wahl (2007) Comparison of ^90^Y-ibritumomab tiuxetan and ^131^I-tositumomab in clinical practice. J. Nucl. Med. 48: 1767–76. 10.2967/jnumed.107.043489 17942813

[2] Milenic DE, Brady ED, Brechbiel MW (2004) Antibody-targeted radiation cancer therapy. Nat. Rev. Drug Discov. 3: 488–99. 10.1038/nrd1413 15173838

[3] (2011) Melphalan with or without Holmium Ho-166 DOTMP Followed by Peripheral Stem Cell Transplantation in Treating Patients with Multiple Myeloma. Available: http://clinicaltrials.gov/ct2/show/NCT00008229?term=Ho166&phase=2&rank=1. Accessed 2011 Nov 1.

[4] (2011) Chemotherapy with or without Strontium-89 in Treating Patients with Prostate Cancer. Available: http://clinicaltrials.gov/ct2/show/NCT00024167?term=Sr89&phase=2&rank=1. Accessed 2011 Nov 1.

[5] Humm JL (1986) Dosimetric aspects of radiolabeled antibodies for tumor-therapy. J. Nucl. Med. 27: 1490–7. 3528417

[6] Zalutsky MR, Pozzi OR (2004) Radioimmunotherapy with alpha-particle emitting radionuclides. Q. J. Nucl. Ned. Mol. Imaging 48: 289–96. 15640792

[7] Kampf G (1988) Induction of DNA double-strand breaks by ionizing radiation of different quality and their relevance for cell inactivation. Radiobiol. Radiother. 29: 631–58. 3253788

[8] Hall EJ, Giaccia A (2006) Radiobiology for the Radiologist. 6th ed. Philadelphia: Lippincott Williams & Wilkins. 546 p.

[9] Gadbois DM, Crissman HA, Nastasi A, Habbersett R, Wang SK, et al (1996) Alterations in the progression of cells through the cell cycle after exposure to alpha particles or gamma rays. Radiat. Res. 146: 414–24. 8927713

[10] Mulford DA, Scheinberg DA, Jurcic JG (2005) The promise of targeted α-particle therapy. J. Nucl. Med. 46 (Suppl 1)199S–204S. 15653670

[11] Kim Y, Brechbiel MW (2012) An overview of targeted alpha therapy. Tumor Biol. 33: 573–90. 10.1007/s13277-011-0286-y PMC7450491 22143940

[12] McDevitt MR, Ma D, Lai LT, Simon J, Borchardt P, et al (2001) Tumor therapy with targeted atomic nanogenerators. Science 294: 1537–40. 11711678 10.1126/science.1064126

[13] Schwartz J, Jaggi JS, O’Donoghue JA, Ruan S, McDevitt M, et al (2011) Renal uptake of bismuth-213 and its contribution to kidney radiation dose following administration of actinium-225-labeled antibody. Phys. Med. Biol. 56: 721–733. 10.1088/0031-9155/56/3/012 PMC3034478 21220845

[14] Essler M, Gärtner FC, Neff F, Blechert B, Senekowitsch-Schmidtke R, et al (2012) Therapeutic efficacy and toxicity of ^225^Ac-labelled vs. ^213^Bi-labelled tumour-homing peptides in a preclinical muse model of peritoneal carcinomatosis. Eur. J Nucl. Med. Mol. Imaging 39: 602–612. 10.1007/s00259-011-2023-6 22237842

[15] Song H, Hobbs RF, Vajravelu R, Huso DL, Esaias C, et al (2009) Radioimmunotherapy of breast cancer metastases with α-particle emitter ^225^Ac: Comparing efficacy with ^213^Bi and ^90^Y. Cancer Res. 69: 8941–8948. 10.1158/0008-5472.CAN-09-1828 PMC2789180 19920193

[16] Sofou S, Thomas JL, Lin HY, McDevitt MR, Scheinberg DA, Sgouros G (2004) Engineered liposomes for potential α-particle therapy of metastatic cancer. J. Nucl. Med. 45: 253–60. 14960644

[17] Bruland OS, Nilsson S, Fisher DR, Larsen RH (2006) High-linear energy transfer irradiation targeted to skeletal metastases by the alpha-emitter ^223^Ra: adjuvant or alternative to conventional modalities? Clin Cancer Res 12: 6250s–7s. 17062709 10.1158/1078-0432.CCR-06-0841

[18] (2011) FDA Grants Fast Track Designation For Alpharadin For Castration Resistant Prostate Cancer In Patients With Bone Metastases. Available: http://www.medicalnewstoday.com/articles/233215.php. Accessed 2012 Aug 18.

[19] Tabuteau A, Pages M, Livet J, Musikas C (1988) Monazite-like phases containing transuranium elements (neptunium and plutonium). J. Mater. Sci. Lett. 7: 1315–7.

[20] Ewing RC (1975) Am. Mineral. (1975) Crystal chemistry of complex niobium and tantalum oxides. 60: 728–33.

[21] Karioris FG, Gowda KA, Cartz L (1981) Heavy ion bombardment of monoclinic ThSiCXt, ThO2, and monazite. Radiat. Eff. Lett. 58: 1–3.

[22] van Vlerken LE, Vyas TK, Amiji MM (2007) Poly(ethylene-glycol)-modified nanocarriers for tumor-targeted and intracellular delivery. Pharm. Res. 24: 1405–14. 10.1007/s11095-007-9284-6 17393074

[23] McDevitt MR, Sgouros G, Finn RD, Humm JL, Jurcic JG, et al (1998) Radioimmunotherapy with alpha-emitting nuclides. Eur. J. Nucl. Med. 25: 1341–51. 10.1007/s002590050306 9724387

[24] Hifumi H, Yamaoka S, Tanimoto A, Akatsu T, Shindo Y, et al (2009) Dextran coated gadolinium phosphate nanoparticles for magnetic resonance tumor imaging. J. Mater. Chem. 19: 6393–7.

[25] Kolsky KL, Mausner LF (1993) Production of no-carrier-added gold-199 for gold cluster-labeled antibodies. Appl. Radiat. Isot. 44: 553–60. 10.1016/0969-8043(93)90169-b 8472026

[26] Buissette V, Giaume D, Gacoin T, Boilot JP (2006) Aqueous routes to lanthanide-doped oxide nanophosphors. J. Mater. Chem. 16: 529–39.

[27] Meiser F, Cortez C, Caruso F (2004) Biofunctionalization of fluorescent rare-earth-doped lanthanum phosphate colloidal nanoparticles. Angew. Chem. Int. Edit. 43: 5954–7. 10.1002/anie.200460856 15547904

[28] Woodward J, Kennel SJ, Stuckey A, Osborne D (2011) Wall J, et al. LaPO4 nanoparticles doped with actinium-225 that partially sequester daughter radionuclides. Bioconj. Chem 22: 766–76. 10.1021/bc100574f 21434681

[29] Woodward JD, Kennel SJ, Mirzadeh S, Dai S, Wall JS, et al (2008) *In vivo* SPECT/CT imaging and biodistribution using radioactive Cd^125m^Te/ZnS nanoparticles. Nanotechnology 18: 175103.

[30] Kennel SJ, Huang Y, Zeng WB, Stuckey A, Wall J (2010) Kinetics of vascular targeted monoclonal antibody. Curr. Drug Deliv. 7: 428. 10.2174/156720110793566227 20950267

[31] Kennel SJ, Woodward J D, Rondinone A J, Wall J, Huang Y, et al (2008) The fate of MAb-targeted Cd(^125m^Te/ZnS) nanoparticles i*n vivo*. Nucl. Med. Biol. 35: 501–14. 10.1016/j.nucmedbio.2008.02.001 18482688

[32] Vanrooijen N (1989) The liposome-mediated macrophage 'suicide' technique. J. Immunol. Methods 124: 1–6. 10.1016/0022-1759(89)90178-6 2530286

[33] Lasbury ME, Durant PJ, Ray CA, Tschang D, Schwendener R, et al (2006) Suppression of alveolar macrophage apoptosis prolongs survival of rats and mice with pneumocystis pneumonia. Immunol. 176: 6443–53. 10.4049/jimmunol.176.11.6443 16709801

[34] Burdick MJ, Sartor O (2010) Bone-targeted therapy in metastatic prostate cancer: osteoclast inhibitors and bone-seeking radiopharmaceuticals. Drug Discov. Today Ther. Strateg. 7: 23–9.

[35] Jaggi JS, Kappel BJ, McDevitt MR, Sgouros G, Flombaum CD, et al (2005) Efforts to control the errant products of a targeted in vivo generator. Cancer Res. 65: 4888–95. 10.1158/0008-5472.CAN-04-3096 15930310

[36] Mirzadeh S, Kumar K, Gansow OA (1993) The chemical fate of ^212^Bi-DOTA formed by beta-decay of ^212^Pb(DOTA)_2_. Radiochim. Acta 60: 1–10.

